# Photo-biohydrogen production potential of *Rhodobacter capsulatus-*PK from wheat straw

**DOI:** 10.1186/1754-6834-6-144

**Published:** 2013-10-07

**Authors:** Saima Shahzad Mirza, Javed Iqbal Qazi, Quanbao Zhao, Shulin Chen

**Affiliations:** 1 Microbial Biotechnology Laboratory, Department of Zoology, University of the Punjab, 54590 Lahore, Pakistan; 2 Biological Systems Engineering, Washington State University, Bioprocessing and Bioproduct Engineering Laboratory, Pullman, WA, USA

**Keywords:** PNSB, Hydrogen yield, Cellulose, Hydrogen yield and monomeric sugars, Furfural and H_2_, Acetic acid and H_2_

## Abstract

**Background:**

Biotechnological exploitation of lignocellulosic biomass is promising for sustainable and environmentally sound energy provision strategy because of the abundant availability of the renewable resources. Wheat straw (WS) comprising of 75-80% cellulose and hemicellulose is one of widely available, inexpensive and renewable lignocellulosic biomass types. The cellulosic and hemicellulose substrate can be hydrolyzed into monomeric sugars by chemical and/or biological methods.

**Results:**

This study examined comparative potential of dilute acid and pre-ammonia pretreated and enzymatically hydrolyzed wheat straw (WS) for hydrogen production by purple non sulfur bacterium *Rhodobacter capsulatus*-PK. Gas production became noticeable after 14 h of inoculation in WS pretreated with 4% H_2_SO_4_. The detoxified liquid hydrolyzate (DLH) after overliming attained a production level of 372 mL-H_2_/L after 16 h under illumination of 120-150 W/m^2^ at 30 ± 2.0°C. Whereas the non-detoxified acid pretreated hydrolyzate (NDLH) of WS could produce only upto 254 mL-H_2_/L after 21 h post inoculation. Evolution of H_2_ became observable just after 10 ± 2.0 h of inoculation by employing 48 h age inoculum on the WS pretreated with 30% ammonia, hydrolyzed with cellulase 80 FPU/g and β-glucosidase 220 CbU/ml at 50°C. Upto 712 ml/L of culture was measured with continuous shaking for 24 h. The 47.5% and 64.2% higher hydrogen volume than the DLH and NDLH substrates, respectively appeared as a function of significantly higher monomeric sugar contents of the enzymatically hydrolyzed substrate and lesser/zero amounts of toxic derivatives including pH reducing agents.

**Conclusion:**

Photofermentative hydrogen production from lignocellulosic waste is a feasible approach for eco-friendly sustainable supply of bioenergy in a cost-effective way. Results of this study provide new insight for addressing biotechnological exploitation of abundantly available and low-cost cellulosic substrates.

## Background

High energy yield without emission of greenhouse gases has rendered hydrogen a clean fuel among all other gaseous fuels [1]. Processes for biological H_2_ production with low energy input and ambient temperature and pressure requirement are highly desirable for the bioenergy sector [2-4]. Purple non sulfur bacteria (PNSB) are group of microbes which produce H_2_ photoheterotrophically under a variety of anaerobic environmental conditions in presence of light and at expense of broad range of substrates [5]. Production cost of biohydrogen can be reduced by employing suitable low cost lignocellulosic feedstocks [6]. Conversion to monomeric sugars of the cellulosic substrate is the first step in such processes. Acid pretreatment at moderate temperature renders hemicelluloses to hydrolyzate of dissolved sugars with acetic acid [7,8].

Enzymatic hydrolysis of the cellulose is typically environmentally friendly and yields no process inhibitory byproduct, but the process is not economically feasible as high enzyme loading is required in order to obtain reasonable yield [9,10]. Pretreatment which alters structure and compositions of lignocellulosic feedstock and makes it more feasible for enzymatic hydrolysis becomes essential in the cellulosic sugar utilization. Removal of lignin and uronic acid substitutes on hemicelluloses, reduction of crystallinity and increase of porosity of the plant cell wall in the pretreatment process can significantly improve accessibility of enzyme to hemicelluloses and cellulose [11]. Lignin acts as a glue to hold cellulose and hemicelluloses together. Among different pretreatment processes, ammonia pretreatment has been proven effective in enhancing efficiency of enzymatic saccharification of lignocellulosic substrates [12,13].

In this study, H_2_ production by enzymatic hydrolysis of ammonia pretreated (delignified) WS was optimized by applying surface response methodology (RSM). The RSM technique is extensively employed for investigation of optimized physiochemical parameters as well as different factors of fermentation media of variety of microbes [14]. Different parameters affecting hydrogen production yield from WS, including inocula age, nitrogen content and amount of substrate were examined. Nitrogenase mediated hydrogen production in purple non sulfur bacteria has also been reported as strongly affected by carbon to nitrogen ratio. Lower carbon to nitrogen ratio slowed down the fermentation process and vice versa. However, presence of excessive fixed nitrogen above critical level in medium repressed nitrogenase activity [15].Further, it was observed that fixed nitrogen is necessary for actively growing cells, especially for H_2_ production at the expense of nitrogenase. This fixed nitrogen must be provided in the form of some favorable nitrogen contents, because many of them have repressive effect on nitrogenase [16]. This study was mainly aimed at developing a systematic tool for predicting hydrogen production from dilute acid treated and enzymatically hydrolyzed wheat straw (WS) in combination with yeast extract. The results add value to the application of WS for the production of clean fuel.

## Results and discussion

In the present study wheat straw was pretreated with dilute H_2_SO_4_ and ammonia prior to enzymatic hydrolysis in separate set of experiments, according to protocols described by different authors in order to obtain more sugar contents available for H_2_ production [13,17,18]. Chemical and elemental compositions of untreated WS are shown in Tables 1 and 2, respectively. It can be seen from Table 2 that the WS contained mainly glucan 37.23% and xylan 21.9%.

**Table 1:** Elemental composition of Wheat Straw (WS) on dry solid basis

**Element**	**Content (μg/g)**	**Element**	**Content (μg/g)**
Ca	2300 ± 0.54	K	12000 ± 0.50
Mg	600 ± 0.27	Na	800 ± 0.30
P	470 ± 0.25	S	860 ± 0.25
As	< 16	Ba	25.0 ± 0.03
Cd	< 0.4	Co	<0.12
Cr	< 2	Cu	1.9 ± 0.01
Fe	71 ± 0.30	Mn	26 ± 0.30
Mo	< 2	Ni	< 2
Pb	< 4	V	< 0.4
Zn	3.3 ± 0.1		

C% (on dry basis) = 48 ± 0.39;

N% (on dry basis) = 0.34 ± 0.01;

Values are means of three replicates ± S.E.M.

**Table 2:** Chemical composition of wheat straw on dry solid basis

**Composition**	**Dry solid (%, w/w)**	**Ammonia pretreatment**
Glucan	37.23 ± 0.24	37.23 ± 0.24
Xylan	21.9 ± 0.12	21.9 ± 0.12
Arabinan	3.43 ± 0.98	3.43 ± 0.98
Galactan	1.60 ± 0.07	1.60 ± 0.07
Lignin^a^	12.0 ± 0.30	9.6 ± 0.05
Extractive	11.1 ± 0.20	11.1 ± 0.20

^a^ Lignin (%) = acid soluble (%) + acid insoluble (%).

Values are means of three replicates ± S.E.M.

Micromolecular sugars are relatively easy to be assimilated into the hydrogen-producing bacterial cells [19]. Therefore in order to better exploit WS for hydrogen production, effectiveness of pretreatment methods of the substrate was compared.

### Effect of ammonia pretreatment on WS

WS was analyzed after ammonia pretreatment followed by enzyme hydrolysis for sugars and lignin contents (Tables 2 and 3). The results showed 20% removal of lignin following ammonia pretreatment (Table 2). The delignification is in close agreement with the results of Han et al. [6]. Ammonia effectively removes from WS lignin that otherwise hinders the enzymatic access to cellulose [20,21]. Han et al. [6] reported WS pretreatment with 15% (v/v) ammonia before employing cellulase and β-glucosidase for enzymatic hydrolysis of WS with reduced lignin for ethanol production.

**Table 3:** Monosaccharides’ concentrations (g/L) in fermentation broth (unexhausted and exhausted) of wheat straw (WS)

**Monosaccharide**	**Unexhausted Broth**						**Exhausted Broth**					
	**Acid pretreatment (4%)**		**Enzymatic hydrolysis (Substrates loading in grams)**				**Acid pretreatment (4%)**		**Enzymatic hydrolysis (Substrates loading in grams)**			
	**DLH**	**NDLH**	**1.0**	**1.5**	**2.0**	**2.5**	**DLH**	**NDLH**	**1.0**	**1.5**	**2.0**	**2.5**
Glucose	2.01^c^ ± 0.00	2.77^c^ ± 0.04	54.10^b^ ± 1.0	55.03^a,b^ ± 0.43	56.10^a^ ± 0.32	57.10^a^ ± 0.41	0.03^c^ ± 0.01	0.04^c^ ± 0.02	4.54^b^ ± 0.10	5.23^a,b^ ± 0.20	6.45^a^ ± 0.23	7.05^a^ ± 0.45
Xylose	9.05^d^ ± 0.16	11.03^c^ ± 0.32	17.81^b,d^ ± 0.3	18.01^a^ ± 1.01	21.10^a^ ± 0.20	22.10^a^ ± 0.30	0.71^c^ ± 0.30	1.31^c^ ± 0.03	2.63^b^ ± 0.10	3.10^b^ ± 0.02	3.38^b^ ± 0.30	5.10^a^ ± 0.01
Arabinose	4.23^b,c^ ± 0.18	6.25^a^ ± 0.23	1.30 ± 0.10	2.34^c^ ± 1.0	4.20^b,c^ ± 0.10	5.10^b^ ± 0.10	-	0.78^a^ ± 0.28	-	-	1.11^a^ ± 0.05	1.12^a^ ± 0.04
Galactose	5.40^b^ ± 0.047	6.23^a^ ± 0.23	-	-	2.20^a.c^ ± 0.04	2.34^c^ ± 0.11	-	-	-	-	1.10^a^ ± 0.02	1.11^a^ ± 0.02
Lignin	-	-	-	-	-	-	-	-	-	-	-	-
HMF	0.26 ± 0.02	0.59 ± 0.02	-	-	-	-	-	-	-	-	-	-
Furfural	0.00	0.03 ± 0.01	-	-	-	-	-	-	-	-	-	-
Acetic acid	2.34 ± 0.034	0.21 ± 0.12	-	-	-	-	-	-	-	-	-	-

Values are means of three replicates ± S.E.M.

### Effect of dilute H_2_ SO_4_ pretreatment on WS

Highest H_2_ production was observed in 4% dilute H_2_SO_4_ acid treated WS hydrolysate. Chemical composition of the 4% acid treated DLH and NDLH is shown in Table 3. In the present study DLH and NDLH portions of the acid treated WS contained xylose and glucose upto 2.01, 2.77 and 9.05 and 11.03 g/L, respectively in addition to arabinose and galactose. While among the inhibitors acetic acid was prominent. It has been reported by Yu et al. [13] that dilute acid pretreatment yielded 81.6% of the monomeric sugars following most of hemicelluloses’ dissolution in the hydrolysate. Acetic acid is formed via de-acetylation of hemicelluloses [13]. Coupling of the temperature upto 50°C with alkaline pH (upto 10) was intended for occurrence of overliming process. Detoxification of acid treated WS hydrolysate by process of overliming completely removed furfural, while monosaccharides and acetic acid retained in DLH (Table 3). HMF on the other hand was present in low concentration. The process of overliming resulted in degradation of 27.4% glucose, 18.05% xylose, 35.52% arabinose and 13.32% galactose. These results are in close agreement with the findings of Mohagheghi et al. [22] who reported 7-34% loss of xylose.

It was observed in the present study that higher concentrations of H_2_SO_4_ caused increases in the yield of monomeric sugars. Excess acid also increased production of furfural, a consequence of xylose degradation. Pattra et al. [23] reported similar results for concentrated acid treated hydrolysate of sugarcane bagasse. Aguilar et al. [24] while studying the kinetics of acid hydrolysis of sugarcane bagasse also reported higher furfural levels. Adverse effects of furfural on enzymes and inhibition of protein and RNA synthesis damage microorganisms, the agents of fermentations. Acetic acid, furfural and hydroxymethyl furfural are microbial toxicants and thus decrease yield of fermentation products [25-27].

### Effect of enzymatic hydrolysis

Table 3 shows conversion yield of ammonia pretreated wheat straw cellulose into different fermentable sugars. Application of 80 FPU/g of cellulase and 220 CbU/ ml of β-glucosidase could yield upto 57.10 ± 0.41 and 22.10 ± 0.30 g/L hexose and pentose for 2.5 g of wheat straw loading/100 ml in 50 mM acetate buffer, respectively. The enzymatic hydrolysis of wheat straw thus resulted in higher yield than the dilute acid pretreatment of glucose and xylose (Table 3). Conversion ratio of cellulose to sugars has been reported responsive to enzyme dose with slight difference in digestibility. Pretreated WS has been demonstrated to be hydrolyzed efficiently even with low enzyme dosage [6,28,29].

### Hydrogen production experiments

Maximum hydrogen production was observed in 4% sulfuric acid pretreated DLH of wheat straw. Although a portion of the sugars lost during overliming process but this treatment resulted in low level of inhibitors. Consequently, more hydrogen production was recorded when the DLH was employed. Comparatively low H_2_ from NDLH of dilute acid pretreated wheat straw is attributable to higher amounts of the inhibitors. Anam et al. [30] also reported highest reducing sugar contents obtained by 4% (v/w) acid concentration while treating sugarcane bagasse. The author reported glucose and yeast extract as carbon and nitrogen sources, respectively important for growth and maintenance of cells in addition to hydrogen production. Complex fermentation media incorporating starting material of natural origins might be of value for certain fermentation applications. In this study upto 372 ml/L of H_2_ volume after 16 hrs was recorded when the acid pretreated DLH of WS was employed. However, in case of Biebl and Pfennig medium (control-MII) where sodium succinate serves as carbon source and yeast extract as nitrogen source for the photofermentation at 30°C, H_2_ production was recorded upto 119 ml/L.

The results are consistent with the reports indicating that alkaline pretreatment of lignocellulosic biomass followed by enzymatic hydrolysis would ultimately lead to improved yield of biohydrogen because of easier access of enzyme into lignocellulosic biomass due to delignification [31-33].

Regarding importance of nitrogen source, Hakobyan et al. [34] reported employment of yeast extract as efficient nitrogen source for biohydrogen production. L-glutamte has been mentioned as most preferred nitrogen source by most of hydrogen producing PNS bacteria [35,36]. For industrial applications, however, other supplements should be investigated, in order to reduce the operational cost and to improve the product yield.

Optimization of parameters like temperature, initial substrate concentration and inoculum age by employing Central Composite Design (CCD) experiments using response surface methodology (RSM) was proved to be an optimum tool. Inoculum age, nitrogen deficit conditions and substrate concentration have been considered as critical parameters for H_2_ production [37,38]. Details of model obtained from the present study with measured response values in terms of hydrogen production as a function of each corresponding statistical treatment combinations of test variables are summarized in Tables 4, 5 &6. The following regression equation of application of RSM resulted in an empirical relationship:

**Table 4:** Coded values of the variables for the central composite design

		**Actual values of coded levels**				
**Variables**	**Coded symbol**	-1	0	1	-1.6818	1.6818
-Inoculum age (h)	X_1_	24	48	72	8.00	88
Nitrogen content (mg)	X_2_	200	300	400	132	468
Substrate (g)	X_3_	1.5	2.0	2.5	1.16	2.84

**Table 5:** **Central composite design (CCD) matrix of three independent variables for H**_**2 **_**production in actual values with experimental results of WS by employing *Rhodobacter capsulatus*-PK (SS-8) in the presence of tungsten lamp with light intensity of 120-150 W/m**^**2**^

**Run No**	**Variables**			**Response**	**Yield**
	**X**_**1**_	**X**_**2**_	**X**_**3**_	**Y hydrogen production (ml)**	**Mole of H**_**2**_**/mole of substrate**
1	-1.000	-1.000	-1.000	528	0.98
2	-1.682	0.000	0.000	587	1.09
3	1.000	-1.000	-1.000	597	1.11
4	0.000	0.000	0.000	709	1.32
5	1.682	0.000	0.000	603	1.12
6	0.000	0.000	0.000	709	1.32
7	0.000	0.000	0.000	712	1.32
8	0.000	0.000	0.000	711	1.32
9	1.000	-1.000	1.000	561	1.04
10	0.000	1.682	0.000	598	1.11
11	0.000	0.000	1.682	551	1.02
12	0.000	0.000	-1.682	559	1.04
13	0.000	0.000	0.000	709	1.32
14	0.000	-1.682	0.000	554	1.03
15	0.000	0.000	0.000	709	1.32
16	1.000	1.000	1.000	542	1.01
17	-1.000	1.000	-1.000	568	1.06
18	-1.000	1.000	1.000	572	1.06
19	1.000	1.000	-1.000	540	1.00
20	-1.000	-1.000	1.000	527	0.98

**Table 6:** Analysis of Variance (ANOVA) for the quadratic model

**Source**	**SS**	**DF**	**MS**	***F*value**	**Probability (*P*) >** ***F***
model	97366.4	9	10818.4	93.93	< 0.0001
Residual (error)	1151.80	10	115.18		
Lack of fit	1142.97	5	228.59	129.39	
Pure error	8.83	5	1.77		
Total	98518.2	19			

Coefficient of determination (*R*^2^) = 0.98; adjusted *R*^2^ = 0.97; coefficient of variation (CV) = 1.77%; SS, sum of squares; DF, degree of freedom; MS, mean square.

Accordingly:

(1) $$\begin{array}{c} Y=\beta_{0}+\beta_{1}X_{1}+\beta_{2}X_{2}+\beta_{3}X_{3}+\beta_{11}X_{1}^{2}+\beta_{22}X_{2}^{2}\\ \;+\beta_{33}X_{3}^{2}+\beta_{12}X_{1}X_{2}+\beta_{13}X_{1}X_{3}+\beta_{23}X_{2}X_{3} \end{array}$$

Here *Y* represents predicted response, *β*_0_ the constant coefficient, *β*_1_, *β*_2_, and *β*_3_ the linear coefficients, *β*_11_, *β*_22_*,* and *β*_33_ the quadratic coefficients, *β*_12_, *β*_13_, and *β*_23_ the cross products coefficients and *X*_1_, *X*_2_ and *X*_3_ were input variables (inoculum age, nitrogen content and substrate loading). From Eq. (1), it can be concluded that in total 20 runs are required for optimized response (Table 5). Analysis of variance (ANOVA) was applied for diagnostic checking of appropriateness of proposed model (Table 6). Coefficient of determination R^2^ and adjusted R^2^ express accuracy and general quality of fitting of the above polynomial model. Three dimensional (3D) surface plots of the fitted polynomial equation illustrate individual and interactive effect of factors on the response within the range of central composite design. The optimum region was also identified based on the main parameters in the overlay plot [39].

(2) $$\begin{array}{l} Y=710.25+5.27X_{1}\\ \;+6.08X_{2}-3.26X_{3}-20.12X_{1}^{2}-4.62X_{2}^{2}\\ \;+5.38X_{3}^{2}-43.30X_{1}X_{2}-50.02X_{1}X_{3}-57.44X_{2}X_{3} \end{array}$$

F test verified significance of this model with F value 93.93 and probability (P) > F (0.0001). As coefficient of variation is indicative of degree of precision of all the compared treatments, therefore low CV (1.77%) approved the reliability of the model of the present study. However, R^2^ = 0.98 also added to the accuracy and consistency of the model as the model predicted and observed values agreed well. Tables 6 &7 explains F test values with reference to respective P values for the estimated parameters. Small P values further approved the significance of the model. Straight line of normal probability showed satisfactory normality assumptions. These parameters appear good indicative of prediction for maximum response within described range of variations of the quadratic model.

**Table 7:** Significance of the coefficients of regression

**Model term**	**Parameter estimate**	**Standard error**	***F*value**	***p*value**
b_0_	710.25	4.38		
b_1_	5.27	2.90	3.29	0.0999
b_2_	6.08	2.90	4.38	0.0628
b_3_	-3.26	2.90	1.26	0.2885
b_11_	-20.12	3.79	28.13	0.0003^a^
b_22_	-4.62	3.79	1.49	0.2509
b_33_	5.38	3.79	2.01	0.1870
b_1_b_2_	-43.30	2.83	234.59	<0.0001^*a*^
b_1_b_3_	-50.02	2.83	313.03	<0.0001^*a*^
b_2_b_3_	-57.44	2.83	412.85	<0.0001^*a*^

^*a*^*p* value less than 0.05 indicate significant model terms.

### Effect of variables on the hydrogen production

**Figure 1:**
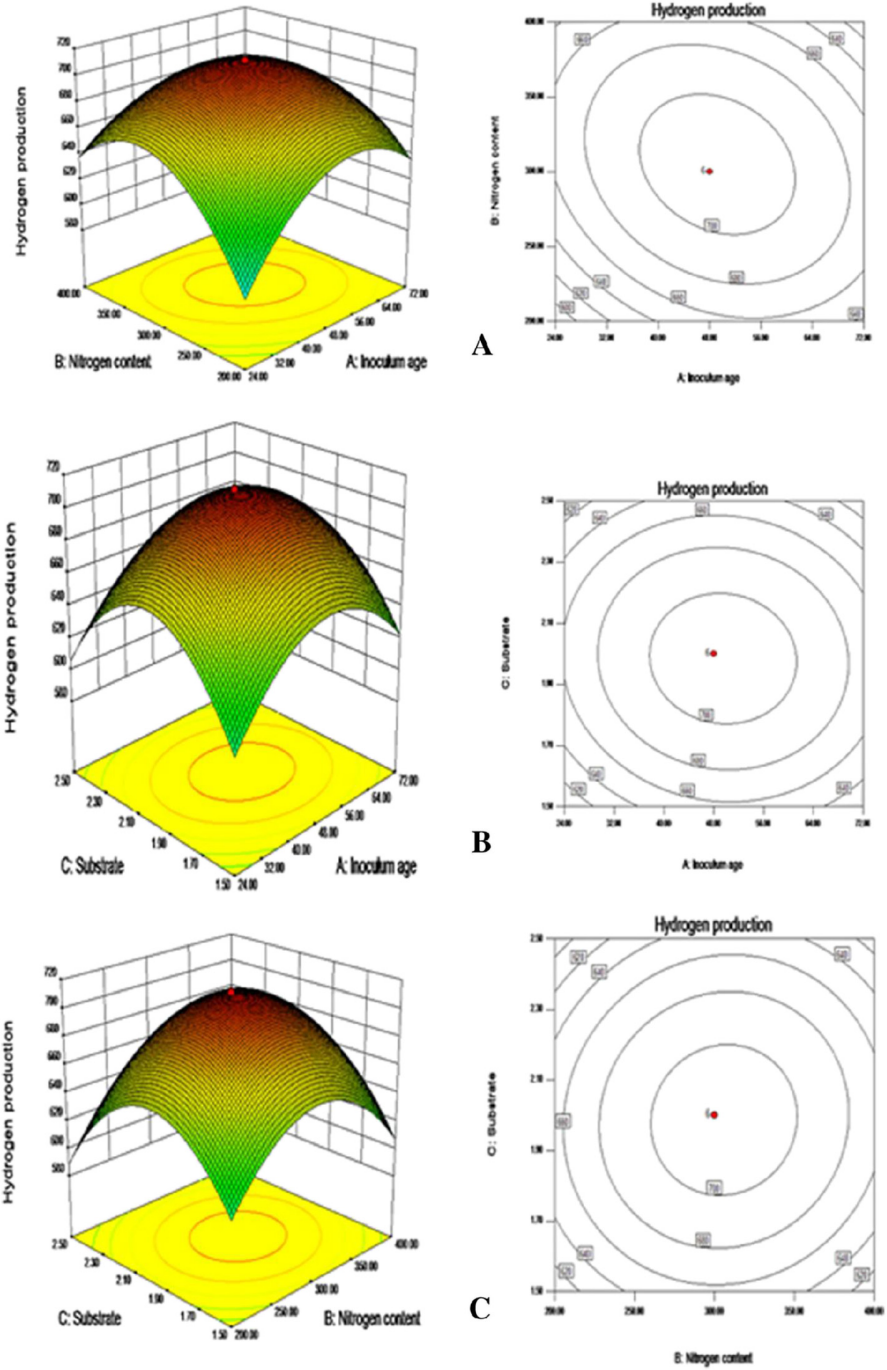
**Response surface and contour plot of H**_**2 **_**production by *Rhodobacter capsulatus*-PK (SS-8) in WS-MII showing combined effects of variables.** i-e nitrogen content and inoculum age **(A)**, substrate amount and inoculum age **(B)** and substrate amount and nitrogen content **(C)**.

Figure 1A shows the effect of inoculum age and nitrogen content on hydrogen production when substrate loading was at their central point. The yield of H_2_ production was low with short inoculum age. Significant improvement in H_2_ production was observed with increases in inoculum age upto 48 h, thereafter the yield decreased with further increments of inoculum age. Basak and Das [40] also reported maximum H_2_ production with 48 h inoculum age. Significant improvement in H_2_ production was also observed initially with increase in nitrogen contents but provision of the substrate beyond 300 mg resulted steady cessation in H_2_ production.

When the substrate loading was increased in this study, increase of inoculum age initially resulted in a significant increase in hydrogen production. At inoculum age of 48 h and substrate loading of 2 g, hydrogen yield reached upto 598 ml/L of culture at fixed nitrogen content. Further increase in substrate loading resulted in decrease of H_2_ production (Figure 1B). Regarding the effect of substrate and nitrogen content on the volume of H_2_ production, it reached upto 607 ml/L when inoculum age was at its central point (Figure 1C). Further increase in substrate concentration and nitrogen content led to gradual decrease in the H_2_ volume. It could be concluded that critical substrate concentration was one of the major factors affecting the conversion rate of enzymatic hydrolysis of cellulose. Qi et al. [12] also reported slow reduction in hydrolysis yield with an increase in substrate concentration. Such reduction of hydrolysis yield has been explained by enzymatic inactivation and decrease in reactivity of cellulosic substrate with proceeding of hydrolysis process [41]. In the present study, hydrogen yield appeared at the expense of monosaccharides. Glucose and xylose were mainly recruited by the bacterium *Rhodobacter capsulatus*- PK for hydrogen production. Table 3 explains the monosaccharides’ concentration in exhausted/fermented broth while H_2_ production was still detectable probably at the expense of acetic acid produced during H_2_SO_4_ pretreatment process. Acetic acid is well recognized substrate for photofermentation by purple non sulfur bacteria [42]. While in case of enzymatic hydrolysis of WS H_2_ production process remained noticeable at slower rate probably at the expense of remaining sugars in fermentation broth (Table 3), because accumulation of acetate was not noticed here.

Xylose and glucose are reported substrates for H_2_ by different microorganisms such as *C. acetobutylicum, C. butyricum* and *Rhodobacter capsulatus,*[43-45]. Lignocellulosic biofuel production is not yet economically competitive with fossil fuels; therefore, successful utilization of all sugars is important for improving the overall economy [46]. It is expected that search of more microbial diversity capable of utilizing maximally the hydrolyzates, ingredients capable/construction of required characteristics possessing GMOs will add to the economics of biohydrogen production from lignocellulogic biomass.

### Confirmation experiments

Prediction of H_2_ production at any tested parameter within the range of experimental design is achieved by employing second–order polynomial regression equation obtained from experimental data.

**Figure 2:**
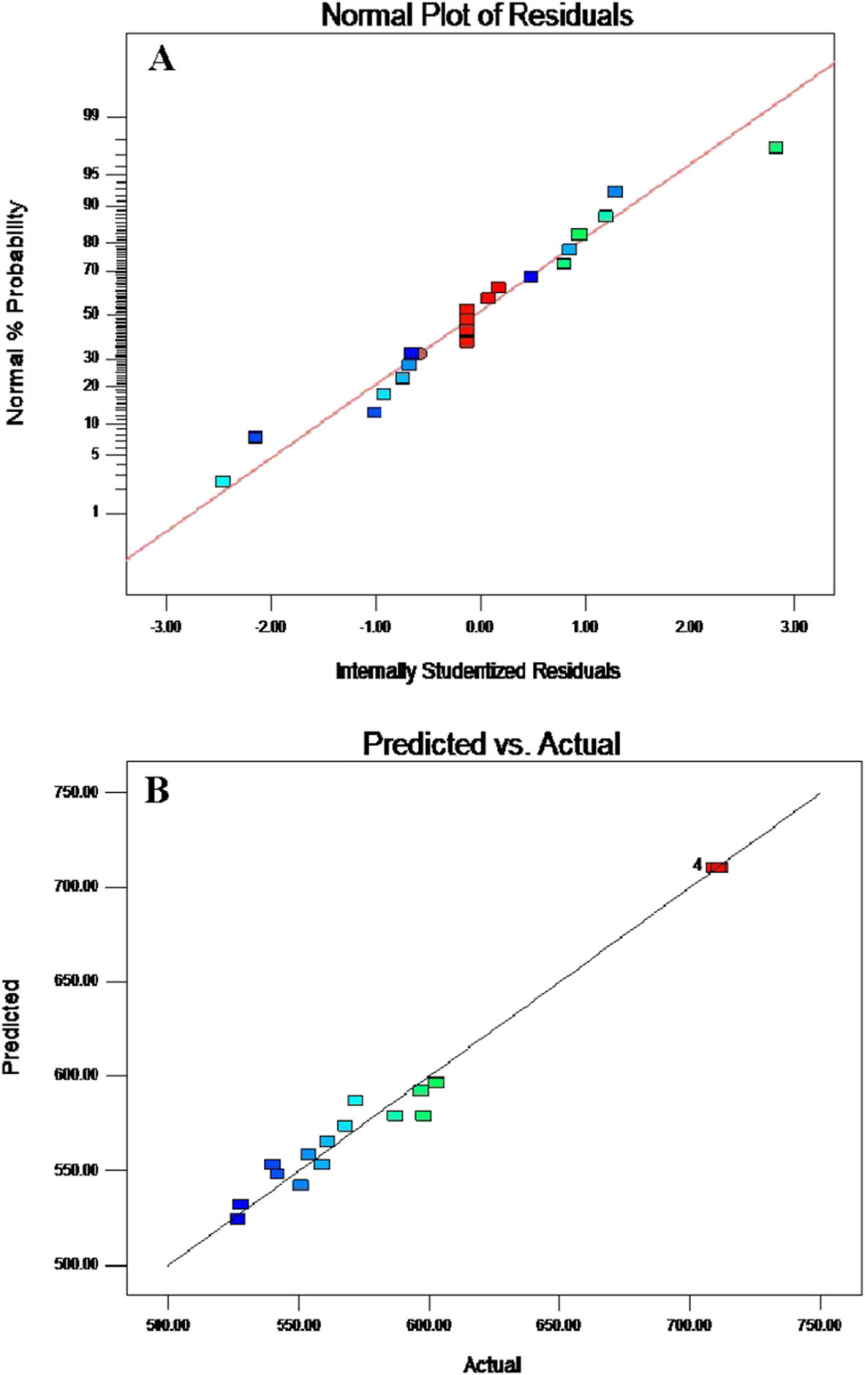
**Response surface for H**_**2 **_**production by *Rhodobacter capsulatus*-PK (SS-8) in WS-MII.** Normal probability of internally studentized residuals **(A)** and observed vs the predicted values **(B)** of CCD.

For better understanding and confirmation of H_2_ production from WS and validity of statistical experimental strategies different confirmation runs were performed (Table 8). Point prediction capability of the software with predicted H_2_ production rate lead to the idea of confirmation of the conditions located within the levels defined previously. The residual and percentage errors were calculated by comparing the actual and predicted H_2_ production rate. It can be seen from values listed in Table 8 that percentage errors between predicted and actual H_2_ production vary from 0.21% to 1.01%. Therefore it can be assumed that developed statistical model is reasonably accurate. Thus central composite design of response surface can be accurately used for prediction and optimization of photofermentative H_2_ production from WS under the experimental conditions employed in this study. The model adequacy can be judged from residual’s least square which is important to ensure for providing maximum approximation on relationship between factors and response when normal probability is checked by normal probability plot of residuals. Straight line in Figure 2A approved the satisfaction of normality assumption. These results are indicative of maximum predictive response with constant variance and quadratic model accuracy Figure 2B.

**Table 8:** **Confirmation experiments of CCD (RSM) model for *Rhodobacter capsulatus*-PK (SS-8) and enzymatically hydrolyzed WS**

**X**_**1**_**(h)**	**X**_**2**_**(mg)**	**X**_**3**_**(g)**	**H**_**2**_**production (ml)**			
			**actual**	**predicted**	**residual**	**error (%)**
48	300	2.00	708.34	710.25	-1.91	0.27
24	300	2.50	611.23	605.61	5.62	0.92
48	400	2.00	673.12	666.31	6.81	1.01
48	300	2.50	651.05	649.55	1.50	0.23
50	320	2.20	697.47	698.94	-1.47	0.21
40	280	1.70	683.09	681.15	1.94	0.28

X_1_ = inoculum age;

X_2_ = nitrogen content;

X_3_ = substrate loading.

Likewise, many attempts have been made by different investigators for identifying suitability of different lignocellulosic substrates as well as process conditions for enhanced hydrogen production. Optimization of some independent process variables (inoculum size, palm oil mill effluent concentration, light intensity, agitation and pH) by response surface methodology (RSM) has been reported by Jamil et al. [47]. Some other parameter’s optimizations like glucose and glutamate concentrations and light intensity have been described by Box-Behnken design of RSM [15]. In several studies lignocellulosic material has been treated chemically and/or biologically for yielding reducing sugars and then employed for photofermentation. Anam et al. [30] reported the photofermentative hydrogen production potential of *Rhodobium marinum* upto 41 ± 16 ml from some reducing sugars obtained from H_2_SO_4_ hydrolysis of sugarcane bagasse. Recently Keskin and Hallenbeck [3] reported potential of 10.5, 8.0 and 14.0 moles of H_2_/mol of substrate from beet molasses, black streep molasses and sucrose, respectively by *Rhodobacter capsulatus* JP91.

## Conclusion

Biohydrogen production was the highest from wheat straw that was enzyme hydrolysed followed by ammonia pretreatment. Yeast extract as nitrogen source, inoculum age and substrate loading for enzyme hydrolysis appeared to affect hydrogen production potential of *Rhodobacter capsulatus*-PK. Acid hydrolysate of the hemicellulose fraction of WS was comparatively less suitable than enzymatically treated hydrolysate due to its comparatively low sugar contents and presence of inhibitors (acetic acid and furfural) for biohydrogen production. According to regression equation of RSM model, the interactions of three tested variables (inoculum age, nitrogen contents and substrate concentration) on H_2_ production from pretreated wheat straw yielded nearly theoretical H_2_ production level. With inoculum age 48 h, nitrogen content of 300 mg/L and 2.0 g/100 mL of substrate loading, the H_2_ volume obtained was 712 ml/L. The model, predicted accurately for maximum photofermentative hydrogen from hydrolysed lignocellulosic biomass.

### Future prospects

Demonstration of biohydrogen yield from local bioresources indicates the feasibility of development of a scalable system for regenerative energy provision employing crop residues. A number of avenues can be pursued to increase yields, including exploration of variety of abundantly available lignocellulosic biomass. Complete utilization of monosaccharides of hydrolysates as well as appropriate adjustment of C/N ratio could affect the efficiency of photofermentative process. Reduction of inhibitors concentrations is also important for the yield improvement. Lignocellulosic biofuel production is not yet economically competitive with fossil fuels; therefore, successful utilization of all sugars is important for improving the overall economy [45]. Other, additional metabolic alterations may be desirable and can be sought from newly developed metabolic flux analysis model of photosynthetic/photoheterotrophic bacterial growth and photofermentation [48]. Finally, light conversion efficiencies might be improved by use of proper light intensities through reduction in the size of the photosynthetic antennae, [49]. It is hoped that like many previously established biotechnological processes, benefiting the humans, inputs from different scientific sectors to this newly growing science would enable existence of a bright future for biofuels with concomitant waste management and provision of renewable and hence cleaner energy sources.

## Materials and methods

### Microorganism

*Rhodobacter capsulatus*-PK was obtained from stock culture collection of Microbial Biotechnology lab., of University of the Punjab, Lahore, Pakistan. The bacterium was isolated from rice paddy field on Biebl and Pfennig medium [50] and has been reported for higher yield of H_2_ following photofermentation in a medium comprised mainly of 2% sugarcane bagasse [51]. The substrate replaced sodium succinate in the original medium described by Biebl and Pfennig [50]. Culture of the bacterium was revived in said medium. The growth was obtained by incubating the inoculated culture bottles at 30 ± 2.0°C for 48 h under illumination of light intensity of 120-150 W/m^2^ and then used as inocula in the subsequent experiments.

**Figure 3:**
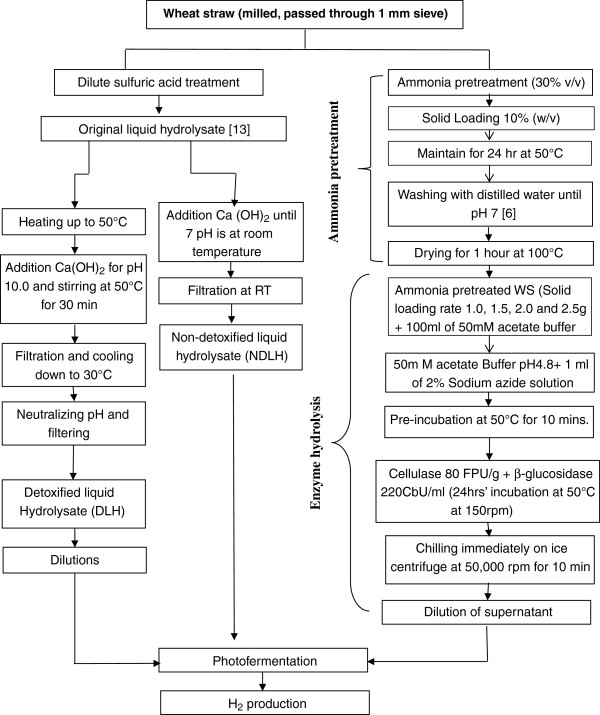
Flow chart indicating two routes for production of hydrogen from lignocellulosic biomass.

Figure 3 explores the whole process from preparing hydrolysate of WS employing dilute sulfuric acid pretreatment and enzymatic hydrolysis to photofermentative production of hydrogen. Wheat straw was washed, air dried and milled before passing through 1 mm sieve which was obtained from Moscow, Idaho, USA. The processed wheat straw was stored in plastic bags for further use at room temperature. Liquid fraction obtained after dilute acid pretreatment of WS was separated via vacuum filtration and divided into two portions. One part was retained as non-detoxified liquid hydrolysate (NDLH) and other was rendered detoxified liquid hydrolysate (DLH) as described in the forthcoming paragraph. Both of the fractions were used as substrates for photofermentative production of hydrogen. While the setup containing Biebl and Pfennig [50] growth medium was used as control run (control-MII).

### Pretreatment of wheat straw with dilute sulfuric acid

Protocol of dilute sulfuric acid pretreatment of wheat straw described by Chen et al. [52] was followed in these experiments. Numeral (v/v) concentrations of sulfuric acid from 1 to 7% were prepared and the wheat straw was suspended and stirred at room temperature in a given dilution at solid loading rate of 10% (w/v). The mixtures were autoclaved at 121°C for 60 min. The autoclaved and cooled liquid hydrolysates were then separated by centrifugation and after vacuum filtration stored at 4°C. The hydrolysate was detoxified by heating upto 50°C with continuous stirring with the help of a stir bar by addition of calcium hydroxide and pH was increased upto 10.0 in the process of overliming. This overlimed mixture was further stirred for 30 min with the help of stirring hot plate. The mixture was then filtered through 0.22 μm membrane (Millipore, MA). The filtrate was allowed to cool upto 30°C and then sulfuric acid was added to attain pH 7. The re-acidified hydrolysate was again filtered through 0.22 μm Millipore filter for removal of precipitate formed. This detoxified liquid hydrolysate was then employed as a fermentation substrate for hydrogen production.

For NDLH calcium hydroxide was added to the acid treated liquid hydrolysate at room temperature to attain 7 pH. Followed filtration through 0.22 μm Millipore filter the liquid served as respective fermentation substrate.

### Enzyme hydrolysis

For this experiment WS was treated with 30% (v/v) ammonia for 24 hour at 50°C with solid loading of 10% (w/v) followed by washing with deionized water until pH became 7. The substrate was then dried in oven at 100°C for one hour for ammonia removal. Thus pretreated WS was subjected to enzyme hydrolysis at solid loading rate of 1.0, 1.5, 2.0 and 2.5 g/100 ml in 50 mM acetate buffer (pH 4.8). In each of the mixture 1 ml of 2% sodium azide solution was added to avoid microbial contamination and then incubated for 10mins at 50°C and 150 rpm. Enzymatic hydrolysis was carried out with novozymes cellulase and β glucosidase of 80 FPU/g and 220 CbU/ml activities, respectively by incubating the reactants at 50°C, 150 rpm for 24 hrs. At termination of the enzymatic hydrolysis a given mixture was chilled immediately on ice and centrifuged at 5000 rpm for 10 min. Then mixture of 20% and 40% of the enzymes and each of acid treated (detoxified and non-detoxified) hydrolysate, respectively was used in original growth medium (Biebl and Pfennig medium) as fermentation substrate. However, in the present study yeast extract upto 300 mg/L was employed as nitrogen source in order to make the medium nitrogen limited. In this study dilutions were made in order to lighten the color of both categories of the hydrolysats for effective absorbance of light in fermentation culture vessels as also reported by Zhu et al. [53], Eroglu et al. [54], Ozgur et al. [55], Boran et al. [56]. Seed inoculum (10%, v/v) was then added to the culture medium. Overview of the process is shown in flow sheet (Figure 3). All experiments were performed under light intensity of 120-150 W/m^2^ at 30 ± 2.0°C with initial pH 7. While mixture composition and initial concentration of fermentation sugars were given in Table 3.

Hydrogen yield was obtained by dividing the maximum hydrogen production (ml/L) by the quantity of substrate (sugars g/L) in the medium [57]. However, for cumulative hydrogen volume, it is described here that at each observational period all the amount of gases produced including H_2_ within the headspace of the batch culture fermenter was removed for analysis.

Response surface methodology (RSM) was used to explore an approximate functional relationship among three-level factorial design variables (Tables 4 &5) and response by using Design Expert software (Central Composite Design Expert Version 8.0.3, Statease, Minneapolis, USA) [58]; Eq. 1.

### Analytical methods

H_2_ production was measured by using Gas chromatograph (GC, CP-3800, Varian, and Walnut Creek, CA) equipped with thermal conductivity detector. Nitrogen was employed as carrier gas and column used was HayeSep Q 80/100 Mesh Silcosteel.

Elemental contents of WS were analyzed with the help of elemental analyzer from Analytical Science Laboratory, Holm Research Center, College of Agriculture and Life Sciences, University of Idaho, Idaho, USA by means of combustion method (Combustion, ASA 29–2.2). One gram of WS was extracted by using the ASTM method [59]. Extractable contents were determined gravimetrically, while glucan, xylan, acid soluble and insoluble lignin were measured according to NREL procedure LAP-002 [60].

Different sugar contents like arabinose, galactose, glucose and xylose in treated and untreated WS were analyzed with the help of ion exchange chromatography system (Dionex ICS-3000) equipped with a CarboPac TM PA 20 (4 × 50 mm) analytical column and CarboPac TM PA 20 (3 × 30 mm) guard column (Dionex Corporation, CA). Membrane filtered (0.22 μm) samples were subsequently eluted isocratically with 0.01 M NaOH after injection at a flow rate of 0.5 ml/min. In a pulsed amperiometric detector analytes were detected and quantified against standard curves by electrochemical detection.

Different inhibitors produced in the hydrolysate were analyzed with high performance liquid chromatography, equipped with Biorad Aminex HPX-87H column (Bio-Rad Laboratories, CA) and refractive index detector according to Sluiter et al. [61]. While mobile phase was 0.005 M sulfuric acid at a flow rate of 0.6 mL/min. Acid treated hydrolysate with more sugar contents and less concentration of inhibitors was selected for hydrogen production experiment.

## Competing interests

The authors declare that they have no competing interests.

## Authors’ contributions

SSM participated in the conception, design, all experimental work, data collection and analysis, and drafted the manuscript. JIQ participated in the critical discussion and manuscript revision. QZ helped in mathematical modeling and SC participated in the critical discussion of the results. All authors read and approved the final manuscript.
